# Effects of inflammation on the kynurenine pathway in schizophrenia — a systematic review

**DOI:** 10.1186/s12974-020-1721-z

**Published:** 2020-02-15

**Authors:** Bruno Pedraz-Petrozzi, Osama Elyamany, Christoph Rummel, Christoph Mulert

**Affiliations:** 1 Center of Psychiatry, Justus-Liebig University, Klinikstrasse 36, Giessen, 35392 Hessen Germany; 2 Giessen Graduate School for Life Sciences, Justus-Liebig University, Leihgesterner Weg 52, Giessen, 35392 Hessen Germany; 3 Alexandria University, 22 El-Guish Road, Alexandria, 21526 Alexandria Egypt; 4 Institute of Veterinary Physiology and Biochemistry, Justus-Liebig University, Frankfurter Strasse 100, Giessen, 35392 Hessen Germany; 5 Center for Mind, Brain and Behavior (CMBB), Hans-Meerwein-Strasse 6, Marburg, 35043 Hessen Germany; 6 Collaborative Research Center 936 (SFB936) - Project C6 - Third Funding Period, Justus-Liebig University, Klinikstrasse 36, Giessen, 35392 Hessen Germany

**Keywords:** Schizophrenia, Inflammation, Kynurenine, Kynurenic acid, Glutamic acid

## Abstract

**Background:**

In the last decade, there has been growing evidence that an interaction exists between inflammation and the kynurenine pathway in schizophrenia. Additionally, many authors found microglial activation in cases of schizophrenia due to inflammatory mechanisms related mostly to an increase of pro-inflammatory cytokines. In order to gain new insights into the pathophysiology of schizophrenia, it is important to incorporate the latest published evidence concerning inflammatory mechanisms and kynurenine metabolism. This systematic review aims to collect reliable recent findings within the last decade supporting such a theory.

**Methods:**

A structured search of electronic databases was conducted for publications between 2008 and 2018 to identify eligible studies investigating patients with schizophrenia/psychosis and the relationship between inflammation and kynurenine pathway. Applicable studies were systematically scored using the NIH Quality Assessment Tools. Two researchers independently extracted data on diagnosis (psychosis/schizophrenia), inflammation, and kynurenine/tryptophan metabolites.

**Results:**

Ten eligible articles were identified where seven studies assessed blood samples and three assessed cerebrospinal fluid in schizophrenic patients.

Of these articles:

- Four investigated the relationship between immunoglobulins and the kynurenine pathway and found correlations between IgA-mediated responses and levels of tryptophan metabolites (i.e., kynurenine pathway).
- Five examined the correlation between cytokines and kynurenine metabolites where three showed a relationship between elevated IL-6, TNF-α concentrations, and the kynurenine pathway.
- Only one study discovered correlations between IL-8 and the kynurenine pathway.
- Two studies showed correlations with lower concentrations of IL-4 and the kynurenine pathway.
- Moreover, this systematic review did not find a significant correlation between CRP (*n* = 1 study), IFN-γ (*n* = 3 studies), and the kynurenine pathway in schizophrenia.

**Interpretation:**

These results emphasize how different inflammatory markers can unbalance the tryptophan/kynurenine pathway in schizophrenia. Several tryptophan/kynurenine pathway metabolites are produced which can, in turn, underlie different psychotic and cognitive symptoms via neurotransmission modulation. However, due to heterogeneity and the shortage of eligible articles, they do not robustly converge to the same findings. Hence, we recommend further studies with larger sample sizes to elucidate the possible interactions between the various markers, their blood vs. CSF ratios, and their correlation with schizophrenia symptoms.

## Introduction

Schizophrenia is a chronic disease characterized mostly by psychotic symptoms, cognitive impairment, and functional decline [1]. To date, the pathophysiology of this psychiatric condition remains unclear. However, research on inflammatory factors in schizophrenia has noticeably grown over the last decade [2].

In this context, the vulnerability-stress-inflammation model has been supported by growing evidence [3, 4]. The model implies that genetic makeup predisposes the subject to be easily affected by stress, which would show inflammatory responses later in life [5]. A review by Lipner et al. has concluded that prenatal maternal stress (e.g., prenatal infection/inflammation, decreased fetal growth, hypoxia-related obstetric complications) has been linked to the development of schizophrenia in offspring [6].

Different animal models have indeed shown correlations with pro-inflammatory cytokines and schizophrenia [7–11]. In addition, authors support immune imbalance in schizophrenia, pointing out a predominance of pro-inflammatory mechanisms [12]. In this case, the presence of different pro-inflammatory metabolites and the inhibition of anti-inflammatory factors (e.g., PGJ2) are demonstrated in the onset and chronic phases of schizophrenia [13–15].

From this standpoint, psychotic episodes in schizophrenia coincide with inflammatory mechanisms linked to the hypothalamic-pituitary stress-inflammatory pathways. This eventually leads to microglial and astrocyte activation [16, 17]. Interestingly, the latter is also associated with the activation of the kynurenine pathway and increased the production of kynurenic acid (KYNA) in the cerebrospinal fluid (CSF) [18, 19]. This metabolite is the only known natural *N*-methyl-d-aspartate (NMDA) receptor antagonist involved in inflammatory processes [20]. This results in the antagonism of the glutamatergic system, which in turn could lead to the dysregulation of dopaminergic neurons. Such changes can be attributed to inflammatory activation [21, 22].

**Fig. 1:**
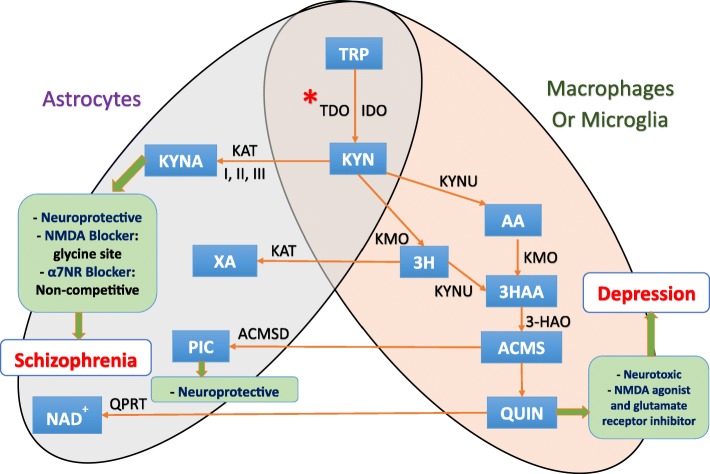
Kynurenine pathway and tryptophan metabolism in the central nervous system. In the central nervous system (CNS), the kynurenine pathway starts by the conversion of tryptophan into kynurenine by indoleamine 2,3-dioxygenase 1 (IDO1), IDO2, or tryptophan 2,3-dioxygenase (TDO). Astrocytes can express both types of enzymes while microglia express only IDO [18, 23]. To a lesser extent, some neurons also possess IDO and/or TDO producing a minor portion of kynurenine [18]. Therefore, kynurenine is available in the CNS via the enzymatic activity of astrocytes, microglia, and some neurons as well as the kynurenine being actively transported into the brain by the large neutral amino acid transporter [19]. Next, kynurenine can follow either of two metabolic branches. First, it can be metabolized into kynurenic acid (KYNA) via kynurenine aminotransferase (KAT) [19, 24, 25] in astrocytes mainly [26] and neurons through irreversible transamination by KAT [27]. The other branch leads to the formation of quinolinic acid (QUIN) exclusively in both microglia and infiltrating macrophages. Both can express kynurenine 3-monooxygenase (KMO) which is absent in human astrocytes [28]. However, both astrocytes and neurons can further catabolize QUIN, produced by neighboring microglial cells, by the enzyme quinolinate phosphoribosyltransferase (QPRTase) [23]. They can also form the neuroprotective picolinic acid (PIC) as they express the enzyme aminocarboxymuconate semialdehyde decarboxylase (ACMSD) [27]. Molecules: 3HAA, 3-hydroxyanthranilic; 3HK, 3-hydroxy-kynurenine; AA, anthranilic acid; ACMS, 2-amino-3-carboxymuconate semialdehyde; KYN, kynurenine; KYNA, kynurenic acid; NAD+, nicotinamide adenine dinucleotide; PIC, picolinic acid; QUIN, quinolinic acid; TRP, tryptophan; XA, xanthurenic acid. Enzymes: 3-HAO, 3-hydroxyanthranlic acid oxygenase; ACMSD, aminocarboxymuconate semialdehyde decarboxylase; IDO, indoleamine 2,3-dioxygenase; KAT, kynurenine aminotransferase; KMO, kynurenine 3-monooxy-genase; KYNU, kynureninase; QPRT, quinolinic acid phosphoribosyltransferase; TDO, tryptophan-2,3-dioxygenase. *Excitation (examples): tryptophan, T-lymphocytes A4, IFN-α, IFN-β, IFN-γ, TNF α. *Inhibition (examples): IL-4, Th2 immunity response, antidepressants, antipsychotics

In the central nervous system (CNS), the kynurenine pathway starts by the conversion of tryptophan into kynurenine by indoleamine 2,3-dioxygenase 1 (IDO1), IDO2, or tryptophan 2,3-dioxygenase (TDO) (Fig. 1). Astrocytes can express both types of enzymes while microglia express only IDO [18, 23]. To a lesser extent, some neurons also possess IDO and/or TDO producing a minor portion of kynurenine [18]. Therefore, kynurenine is available in the CNS via the enzymatic activity of astrocytes, microglia, and some neurons. As well, kynurenine is actively transported into the brain by the large neutral amino acid transporter [19].

Next, kynurenine can follow either of two metabolic branches. First, it can be metabolized into KYNA via kynurenine aminotransferase (KAT) [19, 24, 25] in astrocytes mainly [26] and neurons through irreversible transamination by KAT [27]. The other branch leads to the formation of quinolinic acid (QUIN) exclusively in both microglia and infiltrating macrophages. Both can express kynurenine 3-monooxygenase (KMO) which is absent in human astrocytes [28]. However, both astrocytes and neurons can further catabolize QUIN, produced by neighboring microglial cells, by the enzyme quinolinate phosphoribosyltransferase (QPRTase) [23]. They can also form the neuroprotective picolinic acid (PIC) as they express the enzyme aminocarboxymuconate semialdehyde decarboxylase (ACMSD) [27].

Considering the previously mentioned discoveries, it is important to integrate inflammatory mechanisms and kynurenine metabolism to gain further insights into the pathophysiology of schizophrenia (i.e., pro-inflammatory cytokines that stimulate the production of KYNA in schizophrenia) [19, 29–31]. In mood disorders, for example, there already exists evidence for correlations between both the kynurenine metabolic pathway and inflammation. One example is bipolar disorder, where microglia are shown to be overactive and levels of KYNA were elevated in the CSF [32–34]. Another example is depression, where patients showed decreased levels of KYNA, QUIN, and kynurenine, correlating with pro-inflammatory cytokines [35–37]. However, in schizophrenia, only a few reviews have focused on inflammation and the kynurenine pathway. One example is the review of Wang and Miller, which considered this correlation but in CSF only [37]. Ribeiro-Santos et al. focused on such a correlation concerning cognitive impairment in schizophrenia [38]. Despite the presence of articles where inflammation and the kynurenine pathway in schizophrenia are reviewed, studies that investigate a broader picture or systematic reviews—including serum and CSF for the inflammation markers and the kynurenine pathway—are needed. Indeed, an increasing line of evidence points to such a psycho-neuro-immunology related direction.

To this end, the main aim of this systematic review is to gather state-of-the-art findings in a structured way, based on original research done in the last 10 years on the relationship between inflammation and the kynurenine pathway in schizophrenic patients.

## Methods

### Study selection criteria

Studies of inflammatory cytokines and KYNA in schizophrenia were systematically searched between October and November 2018 using MEDLINE (PubMed, National Center for Biotechnology Information, National Library of Medicine, Maryland, USA), EBSCO (EBSCO Industries Incorporate, Birmingham, AL, USA), Web of Science (Clarivate Analytics, Philadelphia, PA, USA), ProQuest (Cambridge Information Group, Ann Arbor, MI, USA) and SCIELO (Scientific Electronic Library Online, São Paulo Research Foundation, São Paulo, Brasil), Elsevier ScienceDirect (Elsevier Editorials, Amsterdam, The Netherlands), and Cochrane Library (Cochrane, John Wiley and Sons, Hoboken, NJ, USA).

For the article selection criteria, we only considered original research articles. The search was limited to within the last 10 years (i.e., from 2008 to 2018). The inclusion criteria were as follows: (a) observational studies that assess peripheral blood or CSF inflammatory markers, (b) studies that assess schizophrenia/psychosis and kynurenine pathway metabolites, (c) studies with at least 20 subjects in the patient group with their respective matched control group, (d) studies applied in human species, and (e) studies published in the English language. Studies in human species that included any other kind of methodologies (such as interventional studies, meta-analyses, and reviews of any kind) were excluded. Also, animal experimental studies, studies outside the defined time boundaries, studies with too few participants (< 20 patients), and papers not written in English were excluded.

**Fig. 2:**
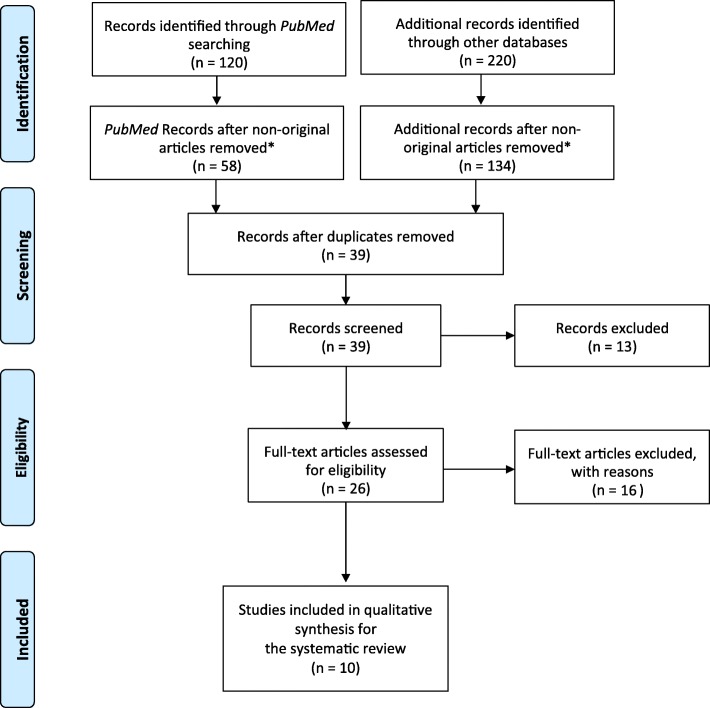
PRISMA flow diagram. *Non-original articles comprise literature reviews, systematic reviews, meta-analyses, and conference abstracts. From Moher et al. [39]

The risk of bias for each included study was assessed according to the quality assessment tools issued by The National Heart, Lung, and Blood Institute (NHLBI). The selected studies are described and summarized in the “Results” section, as well as presented in tables. The process of selection is highlighted in a PRISMA-Chart (Fig. 2). The sociodemographic data and the study design characteristics for each study included were organized in the tables (Tables 1, 2, and 3). Table 4 shows the application of the NHLBI as quality control for the 10 included studies.

**Table 1:** Included articles and their sociodemographic data

Publication	Sample size		Age (years)		Male/female ratio		BMI (kg/m^2^)	
Author, year	Controls	Patients	Controls	Patients	Controls	Patients	Control	Patients
A. Immunoglobulins								
Kanchanatawan et al. 2017 [40]	40	84 clinically stable Sz outpatients	37.4 (12.8)	Without, with physiosomatic symptoms 40.0 (11.1), 41.8 (11.0)	10/30	Without, with physiosomatic symptoms 22/14, 21/22	24.0 (4.3)	Without, with physiosomatic symptoms 24.7 (5.9), 24.5 (4.5)
Kanchanatawan et al. 2018a [41]	40	80 Sz outpatients, 40 deficit, and 40 non-deficit patients	37.9 (12.8)	Non-deficit 41.3 (10.8), deficit 40.9 (11.9)	10/30	Non-deficit 22/18, deficit 21/19	24.0 (4.3)	Non-deficit 26.0 (5.2), deficit 22.9 (4.6)
Kanchanatawan et al. 2018b [42]	40	80 Sz outpatients, 40 deficit, and 40 non-deficit patients	37.9 (12.8)	Non-deficit 41.3 (10.8), deficit 40.9 (11.4)	10/30	Non-deficit 22/18, deficit 21/19	24.0 (4.3)	Non-deficit 26.0 (5.2), deficit 22.9 (4.6)
Bechter et al. 2010 [43]	4100	Inpatient Sz = 39, inpatient Af = 24	ND	Sz = 33.2 (13.1), Af = 45.4 (9.8)	ND	34/29	ND	ND
B. Interleukins								
Szymona et al. 2017 [44]	45	51 Sz inpatients due to acute relapse at time of admission, after a 4-week treatment and remission	24.2 (5.6)	26.9 (8.2)	23/22	32/19	22.14 ± (5.66)	25.24 ± (4.76)
Barry et al. 2009 [45]	36	34 outpatients (Sz or SzA disorder)	33.7 (6.6)	37.3 (8.9)	26/10	26/8	25 (4.0)	28 (4.3)
Kim et al. 2009 [46]	174	71 acute admitted medication-naïve psychotic patients or medication-free for at least 4 months assessed on admission and Discharge after 6 weeks.	32.49 (10.69)	33.9 (12.2)	78/96	32/39	22.11 (2.9)	23.0 (3.9)
Schwieler et al. 2015 [47]	37	23 Sz outpatients	23.0 (22.0–25.5)*	35.0 (32.0–41.0)*	23/14	15/8	23.0 (22.0–26.0)*	26.2 (22.1–27.2)*
Kegel et al. 2017 [48]	Outpatient (MZ)	Outpatient (DZ)	(MZ)	(DZ)	(MZ)	(DZ)	(MZ)	(DZ)
	12 (2 single twins)	11 (one single twin)	57.0 (6.4)	52.9 (7.5)	7/5	3/8	27.5 (6.9)	29.3 (7.9)
C. C-reactive protein								
Wurfel et al. 2017 [49]	92	Inpatient MDD (*N* = 35), BD (*N* = 53), SzA (*N* = 40), acutely ill Sz (*n* = 21)	32.3 (10.4)	MDD 38.8 (13.8), BD 40.2 (11.0), SzA 39.0 13.0), Sz 38.9 12.9)	33/59	MDD 16/19, BD 16/37, SzA 24/40, Sz 17/4	27 (6)	MDD 30 (9), BD 30 (8), SzA 30 (9), Sz 28 (6)

All age and BMI results are shown as mean (SD) except values indicated by the asterisk symbol, data are represented as median (interquartile range)

*Abbreviations*: *3HK* 3-OH-kynurenine, *AA* anthranilic acid, *Af* affective disorder, *BPRS* the Brief Psychiatric Rating Scale, *CANTAB* The Cambridge Neuropsychological Test Automated Battery, *CDSS* Calgary Depression Scale for Schizophrenia, *CRP* C-reactive protein, *CSF* cerebrospinal fluid, *DSM-IV-TR* Diagnostic and Statistical Manual of Mental Disorders-Text Revision, *DZ* dizygotic twins, *FF* Fibromyalgia and Chronic Fatigue Syndrome Rating Scale, *GAF* Global Assessment of Functioning Scale, *HAM-A* Hamilton Anxiety Rating Scale, *HAM-D* Hamilton Depression Rating Scale, *ICD 10* The International Classification of Diseases Tenth Edition, *IFN-* interferon-, *Ig* immunoglobulin, *IL-* interleukin-, *KYN* kynurenine, *KYNA* kynurenic acid, *MDD* major depressive disorder, *MINI* Mini-International Neuropsychiatric Interview, *MZ* monozygotic twins, *ND* not determined, *PA* picolinic acid, *PANSS* The Positive and Negative Syndrome Scale, *QUIN* quinolinic acid, *SANS* Scale for the Assessment of Negative Symptoms, *SAPS* Scale for the Assessment of Positive Symptoms, *SCID-I/II for DSM-IV* Structured Clinical Interview for Diagnostic and Statistical Manual IV Axis I/II Disorders, *SDS* Schedule for Deficit Syndrome, *sIL-2R* soluble interleukin-2 receptor, *SPQ-B* Schizotypal Personality Questionnaire Brief, *Sz* schizophrenia, *SzA* schizo-affective disorder, *TGF-* transforming growth factor, *TNF­α* tumor necrosis factor­α, *Trp* Tryptophan, *TRYCATs* tryptophan catabolites, *XA* xanthurenic acid, *YMRS* The Young Mania Rating Scale

**Table 2:** Included articles and their study design

Publication	Study design	Country	Clinical scales	Investigation	
Author, year				Medium	Markers
A. Immunoglobulins					
Kanchanatawan et al. 2017 [40]	Observational cross-sectional with healthy controls	Thailand	DSM IV, FF, PANSS, SANS, SDS, HAM-D, HAM-A, CANTAB	Blood	3HK, KYNA, QUIN, AA, XA, PA, and IgA and IgM responses to these six TRYCATs
Kanchanatawan et al. 2018a [41]	Observational cross-sectional with healthy controls	Thailand	DSM-IV-TR, Thai version of MINI, SDS, SANS, PANSS, BPRS	Blood	3HK, KA, QUIN, AA, XA, PA, and IgA and IgM responses to these six TRYCATs
Kanchanatawan et al. 2018b [42]	Observational cross-sectional with healthy controls	Thailand	DSM-IV, SDS, SANS, PANSS, BPRS	Blood	3HK, KYNA, QUIN, AA, XA, PA, and IgA responses to these six TRYCATs
Bechter et al. 2010 [43]	Observational cross-sectional with healthy controls	Germany	ICD-10	Blood and CSF	KYN, Trp, albumin, IgG, IgA, IgM, oligoclonal IgG
B. Interleukins					
Szymona et al. 2017 [44]	Observational prospective case-control study	Poland	ICD-10 (F20), SAPS, SANS, PANSS	Blood	KYNA, 3-HK, sIL-2R, IFN-α, IL-4
Barry et al. 2009 [45]	Observational cross-sectional with healthy controls	Ireland	DSM-IV, SCID-I for DSM-IV, SAPS, SANS, CDSS	Blood	L-Trp, KYN, 3HK, KYNA, 3-hydroxy-anthranilic acid, XA and 3-nitro-l-tyrosine
Kim et al. 2009 [46]	Observational prospective case-control study	Korea	DSM IV, PANSS	Blood	Trp and KYN as well as IL-2, IL-4, IL-6, TNF-α, IFN-γ, TGF-β_1_
Schwieler et al. 2015 [47]	Observational cross-sectional with healthy controls	Sweden	DSM­IV, BPRS, GAF	CSF	Cytokines: IL­1β, IL­2, IL­4, IL­6, IL­8, IL­10, IL­18, TNF­α, IFN­α­2a, and IFN­γ as well as KYNA, KYN, and Trp
				Human astrocyte culture	Effects of IL-6 on KYN and KYNA
Kegel et al. 2017 [48]	Observational cross-sectional with healthy control twins	Sweden	SCID-I, SCID-II, SANS, SAPS, SPQ-B, HAM-D, YMRS, GAF	CSF	KYNA, QUIN, Trp, IL-6, IL-8, TNF-α, albumin
				Blood	CRP and albumin
C. C-reactive protein					
Wurfel et al. 2017 [49]	Observational cross-sectional with healthy controls	USA	DSM-IV, HAM-D, YMRS, BPRS version 4.0, CORE assessment of psychomotor change	Blood	High-sensitivity CRP, Trp, KYN, KYNA, 3HK

*Abbreviations*: *3HK* 3-OH-kynurenine, *AA* anthranilic acid, *Af* affective disorder, *BPRS* The Brief Psychiatric Rating Scale, *CANTAB* The Cambridge Neuropsychological Test Automated Battery, *CDSS* Calgary Depression Scale for Schizophrenia, *CRP* C-reactive protein, *CSF* cerebrospinal fluid, *DSM-IV-TR* Diagnostic and Statistical Manual of Mental Disorders-Text Revision, *DZ* dizygotic twins, *FF* Fibromyalgia and Chronic Fatigue Syndrome Rating Scale, *GAF* Global Assessment of Functioning Scale, *HAM-A* Hamilton Anxiety Rating Scale, *HAM-D* Hamilton Depression Rating Scale, *ICD 10* The International Classification of Diseases Tenth Edition, *IFN-* interferon-, *Ig* immunoglobulin, *IL*- interleukin-, *KYN* kynurenine, *KYNA* kynurenic acid, *MDD* major depressive disorder, *MINI* Mini-International Neuropsychiatric Interview, *MZ* monozygotic twins, *ND* not determined, *PA* picolinic acid, *PANSS* The Positive and Negative Syndrome Scale, *QUIN* quinolinic acid, *SANS* Scale for the Assessment of Negative Symptoms, *SAPS* Scale for the Assessment of Positive Symptoms, *SCID-I/II for DSM-IV* Structured Clinical Interview for Diagnostic and Statistical Manual IV Axis I/II Disorders, *SDS* Schedule for Deficit Syndrome, *sIL-2R* soluble interleukin-2 receptor, *SPQ-B* Schizotypal Personality Questionnaire Brief, *Sz* schizophrenia, *SzA* schizo-affective disorder, *TGF*- transforming growth factor, *TNF­α* tumor necrosis factor­α, *Trp* Tryptophan, *TRYCATs* tryptophan catabolites, *XA* xanthurenic acid, *YMRS* The Young Mania Rating Scale

**Table 3:** Included articles and their main Findings

Author, year	Sample size		Main findings
	Controls	Patients	
A. Immunoglobulins			
Kanchanatawan et al. 2017 [40]	40	84 clinically stable Sz outpatients	- Physiosomatic symptoms were significantly associated with IgA/IgM responses to TRYCATs, including increased IgA responses to 3 HK, picolinic acid, and xanthurenic acid and lowered IgA responses to QUIN acid and AA
Kanchanatawan et al. 2018a [41]	40	80 Sz outpatients, 40 deficit, and 40 non-deficit patients	- Deficit schizophrenia increased TRYCAT vs. controls and non-deficit - Both schizophrenia subgroups showed increased IgA responses to 3-OH-kynurenine - Negative symptoms positively correlated with increased IgA responses directed against picolinic acid and inversely with anthranilic acid without significant associations between positive ones and IgA responses to TRYCATs
Kanchanatawan et al. 2018b [42]			
Bechter et al. 2010 [43]	4100	Inpatient Sz = 39, inpatient Af = 24	- Intrathecal immune response in 9 patients (4 with schizophrenia) - Blood-brain barrier dysfunction in 18 patients (9 of them with schizophrenia) - Neopterin concentrations increased in 14 patients (8 of them with schizophrenia) - Absence of any significant changes in tryptophan nor kynurenine metabolites
B. Interleukins			
Szymona et al. 2017 [44]	45	51 Sz inpatients	- KYNA/3-HK ratio and IL-4 levels decreased - Negative correlation between IFN-α and 3-HK level - A positive correlation between KYNA and IFN-α
Barry et al. 2009 [45]	36	34 outpatients	- Significant higher kynurenine/tryptophan ratio in patients without significant correlation with IFN-γ values.
Kim et al. 2009 [46]	174	71 medication-free assessed on admission and discharge after 6 weeks	At admission: - Th1-specific IFN-γ and TNF-α, and Th2-related IL-6 were higher - Tryptophan was significantly lower - Th1-related IL-2 and Th2-specific IL-4 were significantly lower - Th1/Th2 ratio ([IFN-γ]/[IL-4]) correlated positively with the tryptophan breakdown index ([kynurenine]/plasma [tryptophan]) After six weeks of treatment: - A significant reduction of plasma IL-6 and TNF- α - Both mean tryptophan and kynurenine levels were significantly increased
Schwieler et al. 2015 [47]	37	23 Sz	- Correlation between IL-6 and KYNA-production
Kegel et al. 2017 [48]	Outpatient (MZ)	Outpatient (DZ)	- Correlation between IL-8 and QUIN - Correlation between TNF-α and QUIN
	12 (2 single twins)	11 (one single twin)	
C. C-reactive protein			
Wurfel et al. 2017 [49]	92	Inpatient MDD (*N* = 35), BD (*N* = 53), SzA (*N* = 40), acutely ill Sz (*n* = 21)	Sz patients versus MDD, BD and SZA subgroups showed no significant difference in KYNA/QUIN and compared to healthy controls

*Abbreviations*: *3HK* 3-OH-kynurenine, *AA* anthranilic acid, *Af* affective disorder, *BPRS* The Brief Psychiatric Rating Scale, *CANTAB* The Cambridge Neuropsychological Test Automated Battery, *CDSS* Calgary Depression Scale for Schizophrenia, *CRP* C-reactive protein, *CSF* cerebrospinal fluid, *DSM-IV-TR* Diagnostic and Statistical Manual of Mental Disorders-Text Revision, *DZ* dizygotic twins, *FF* Fibromyalgia and Chronic Fatigue Syndrome Rating Scale, *GAF* Global Assessment of Functioning Scale, *HAM-A* Hamilton Anxiety Rating Scale, *HAM-D* Hamilton Depression Rating Scale, *ICD 10* The International Classification of Diseases Tenth Edition, *IFN*- interferon-, *Ig* immunoglobulin, *IL*- interleukin-, *KYN* Kynurenine, *KYNA* kynurenic acid, *MDD* major depressive disorder, *MINI* Mini-International Neuropsychiatric Interview, *MZ* monozygotic twins, *ND* not determined, *PA* picolinic acid, *PANSS* The Positive and Negative Syndrome Scale, *QUIN* quinolinic acid, *SANS* Scale for the Assessment of Negative Symptoms, *SAPS* Scale for the Assessment of Positive Symptoms, *SCID-I/II for DSM-IV* Structured Clinical Interview for Diagnostic and Statistical Manual IV Axis I/II Disorders, *SDS* Schedule for Deficit Syndrome, *sIL-2R* soluble interleukin-2 receptor, *SPQ-B* Schizotypal Personality Questionnaire Brief, *Sz* schizophrenia, *SzA* schizo-affective disorder, *TGF*-transforming growth factor, *TNF­α* tumor necrosis factor­α, *Trp* Tryptophan, *TRYCATs* tryptophan catabolites, *XA* xanthurenic acid, *YMRS* The Young Mania Rating Scale

**Table 4:** Quality assessment for the included studies

Year	Author	Sample Size		Q1	Q2	Q3	Q4	Q5	Q6	Q7	Q8	Q9	Q10	Q11	Q12
		Controls	Patients												
A. Immunoglobulins															
2017	Kanchanatawan et al.	40	84 clinically stable Sz outpatients	Yes	CD	NR	Yes	Yes	Yes	NR	Yes	NA	Yes	NR	Yes
2018a	Kanchanatawan et al.	40	80 Sz outpatients, 40 deficit, and 40 non-deficit patients	Yes	Yes	Yes	Yes	Yes	Yes	No	No	Yes	Yes	NA	Yes
2018b	Kanchanatawan et al.	40	80 Sz outpatients, 40 deficit, and 40 non-deficit patients	Yes	CD	Yes	Yes	Yes	Yes	NR	CD	NA	Yes	Yes	Yes
2010	Bechter et al.	4100	Inpatient Sz = 39, inpatient Af = 24	Yes	Yes	No	Yes	Yes	Yes	No	No	Yes	Yes	NA	No
B. Interleukins															
2017	Szymona et al.	45	51 Sz inpatients due to acute relapse at time of admission, after a 4-week treatment and remission	Yes	Yes	NR	Yes	Yes	Yes	NR	Yes	NA	Yes	Yes	Yes
2009	Barry et al.	36	34 outpatients (Sz or SzA disorder	Yes	Yes	No	Yes	Yes	Yes	No	No	Yes	Yes	NA	Yes
2009	Kim et al.	174	71 acute admitted medication-naïve psychotic patients or medication-free for at least 4 months assessed on admission and discharge after 6 weeks.	Yes	Yes	NR	Yes	Yes	Yes	NR	CD	NA	Yes	Yes	Yes
2015	Schwieler et al.	37	23 Sz outpatients	Yes	Yes	No	Yes	Yes	Yes	No	No	Yes	Yes	NA	Yes
2017	Kegel et al.	Outpatient (MZ)	Outpatient (DZ)	Yes	Yes	No	Yes	Yes	Yes	No	No	Yes	Yes	NA	Yes
		12 (2 single twins)	11 (one single twin)												
C. C-reactive protein															
2017	Wurfel et al.	92	Inpatient MDD (*N* = 35), BD (*N* = 53), SzA (*N* = 40), acutely ill Sz (*n* = 21)	Yes	CD	NR	Yes	Yes	Yes	NR	CD	NA	Yes	Yes	Yes

*Abbreviations*: *CD* cannot determine, *NA* not applicable, *NR* not reported, *Af* affective disorder, *DZ* dizygotic twins, *MDD* major depressive disorder, *MZ* monozygotic twins, *Sz* schizophrenia, *SzA* schizo-affective disorder

Questions: Q1. Was the research question or objective in this paper clearly stated? Q2. Was the study population clearly specified and defined? Q3. Was the participation rate of eligible persons at least 50%? Q4. Were all the subjects selected or recruited from the same or similar populations (including the same time period)? Were inclusion and exclusion criteria for being in the study prespecified and applied uniformly to all participants? Q5. Was a sample size justification, power description, or variance and effect estimates provided? Q6. For the analyses in this paper, were the exposure(s) of interest measured prior to the outcome(s) being measured? Q7. Was the timeframe sufficient so that one could reasonably expect to see an association between exposure and outcome if it existed? Q8. For exposures that can vary in amount or level, did the study examine different levels of the exposure as related to the outcome (e.g., categories of exposure, or exposure measured as continuous variable)? Q9. Were the exposure measures (independent variables) clearly defined, valid, reliable, and implemented consistently across all study participants? Q10. Was the exposure(s) assessed more than once over time? Q11. Were the outcome measures (dependent variables) clearly defined, valid, reliable, and implemented consistently across all study participants? Q12. Were the outcome assessors blinded to the exposure status of participants? Q13. Was loss to follow-up after baseline 20% or less? Q14. Were key potential confounding variables measured and adjusted statistically for their impact on the relationship between exposure(s) and outcome(s)?

Taken from: The National Heart, Lung and Blood Institute. Study Quality Assessment Tools. Available at: https://www.nhlbi.nih.gov/health-topics/study-quality-assessment-tools

### Literature search strategy

Two authors (BPP and OE) independently conducted a systematic literature search in accordance with the PRISMA (Preferred Reporting Items for Systematic Reviews and Meta-analyses) statement [39].

To select articles that fulfill our main research aim and inclusion criteria, three different categories were introduced. For each of those, similar medical subject headings (MeSH) were included to refine the search, obtaining the following: category 1—schizophrenia (“Psychosis” or “Schizophrenia”), category 2—inflammation (“immune response,” “cytokines,” “interleukins,” or “inflammation”), and category 3—kynurenine (“Kynurenine” or “Kynurenic acid”).

Afterwards, the three categories were applied to the search field following this operation: Category 1 (one term) AND Category 2 (one term) AND Category 3 (one term). In this case, we chose one term from each category per each search operation. Hence, the search process resulted in the following Boolean expressions: “Psychosis AND immune response AND Kynurenine”; “Psychosis AND interleukins AND Kynurenine”; “Psychosis AND inflammation AND Kynurenine”; “Psychosis AND cytokines AND Kynurenine”; “Psychosis AND immune response AND Kynurenic acid”; “Psychosis AND interleukins AND Kynurenic acid”; “Psychosis AND inflammation AND Kynurenic acid”; “Psychosis AND cytokines AND Kynurenic acid”; “Schizophrenia AND immune response AND Kynurenine”; “Schizophrenia AND interleukins AND Kynurenine”; and “Schizophrenia AND inflammation AND Kynurenine”.

## Results

### Description of the studies

Out of 340 candidate publications, our search generated 10 publications, which were included for a qualitative synthesis (Fig. 2). All of the included studies were observational (*n* = 918). Eight followed a cross-sectional case-control methodology, whereas only two applied a prospective case-control longitudinal method [44, 46]. The dataset comprised of 918 unique patients from 7 countries (Table 2). The sociodemographic information regarding each study is reported in Table 1. The characteristics, study design, and main findings for each study are reported in Tables 2 and 3.

Eight studies (80%) used the Diagnostic and Statistical Manual of Mental Disorders (DSM) criteria to define schizophrenia, and the other two (20%) considered International Classification of Diseases 10th Version (ICD-10) criteria. Two studies reported additional clinical evaluations by a board-certified psychiatrist involving expert judgment [48, 49].

The patients’ exclusion criteria included the following conditions: autoimmune disorders, comorbid psychiatric disorders (mostly substance abuse/dependence), learning disability, use of immunomodulatory non-psychotropic drugs (such as corticosteroids), acute infectious reactions or diseases, allergies, inflammatory response prior to the assessment, leukopenia, neurological disorders (meningoencephalitis, multiple sclerosis, vascular disorders, head injury, epilepsy, Parkinson’s disease, Alzheimer’s disease, and Huntington’s disease), cancer, diabetes mellitus (type 1 and 2), cardiovascular disorder (such as vascular hypertension or past myocardial infarction), inflammatory bowel disease, chronic obstructive pulmonary disease, and finally use of antioxidants and omega 3 (ω3) polyunsaturated fatty acids.

Only 4 out of 10 studies described controls’ exclusion criteria precluding the following conditions: personal or family history of psychiatric illness, autoimmune disease, substance abuse/dependence, medical conditions, or concomitant medications likely to influence CNS or immunological function including cardiovascular, respiratory, endocrine, and/or neurological diseases [45, 46, 48, 49]. Only one study disregarded the exclusion of patients with comorbid medical conditions, arguing that their frequency is significantly elevated in a severely ill psychiatric population [49].

Seven out of 10 studies have performed peripheral blood analysis, whereas 3 out of 10 studies have tested CSF. Interferon (IFN-) γ, IL-2, IL-4, and tumor necrosis factor alpha (TNF-α) have been measured as inflammatory substances within three publications [44, 46–48]. Other inflammatory substances included IL-6, IL-8, IL-10, IL-18, IFN-α-2a, IFN-α, IL-1β, C-reactive protein (CRP), immunoglobulin (Ig) M, IgA, and CSF cell counts.

Authors used enzyme-linked immunosorbent assay (ELISA) for the assessment of inflammatory markers. In special conditions when high-sensitive (hs) CRP or some specific markers were measured, authors performed special methodology, for instance, immunoturbidimetry of CRP [49]. High-performance liquid chromatography (HLPC) was the standard method to measure kynurenine and tryptophan metabolites. Variations between studies exclusively depend on technical aspects as well as devices used for analysis.

To assess serum markers, fasting venous blood samples were collected in the morning. Afterwards, the blood samples were centrifuged and stored at − 70 °C. Within the three studies analyzing CSF samples, the authors applied centrifugation, cryopreservation, and ELISA analysis. The intra- and inter-assay coefficients between studies were similar. Detection limits were reported and adequate for respective analyses.

### Blood immunoglobulins and kynurenine pathway deregulation in schizophrenia

Three included studies approached immunoglobulins and the kynurenine pathway deregulation in the serum of schizophrenic patients. For the two of them, information was obtained from one large study divided between two publications. One study classified schizophrenic patients according to the Schedule for the Deficit Syndrome (SDS) into two groups: patients with deficit schizophrenia (*n* = 40) and those with non-deficit schizophrenia (*n* = 40). The authors tried to delineate the differences in tryptophan catabolites’ (TRYCATs) profile between both groups in comparison with healthy controls (*n* = 40). Deficit schizophrenia was accompanied by an activated TRYCATs pathway when compared to controls and the non-deficit schizophrenia subgroup [41, 42]. Moreover, both schizophrenia subgroups showed increased IgA responses to 3-OH-kynurenine as compared to controls while KYNA and anthranilic acid (AA) were relatively lowered in the combined schizophrenia groups versus controls with respect to increased levels of noxious TRYCATs [41, 42].

The non-deficit schizophrenia patient subgroup versus healthy controls was characterized by relatively increased PIC but lowered QUIN levels [41, 42].

Interestingly, deficit schizophrenia showed much higher IgA responses to PIC, xanthurenic acid (XA), and QUIN; an increased QUIN/KYNA ratio; relatively lowered AA and KYNA levels; and lowered IgA responses to both acids as compared to non-deficit schizophrenia [41, 42].

The negative symptoms of schizophrenia are significantly and positively correlated with increased IgA responses directed against PIC and inversely with AA, whereas no significant associations between positive symptoms and IgA responses to TRYCATs were found [41, 42].

Another study reported an association between physiosomatic (PS) symptoms and IgA-mediated response in patients with schizophrenia (*n* = 84) in comparison with controls (*n* = 40). PS symptoms, in this case, covers unexplained somatic symptoms (i.e., fatigue, muscle tension, flu-like malaise) related mostly with anxiety and depression. These PS symptoms, which were elevated in more than 50% of the patients, were significantly associated with IgA/IgM responses to TRYCATs, including increased IgA responses to 3 HK, PIC, and XA and lowered IgA responses to QUIN and AA [40].

### CSF immunoglobulins and kynurenine pathway deregulation in schizophrenia

Bechter and colleagues [43] analyzed CSF samples from 63 hospitalized patients with schizophrenia (*n* = 39) and affective disorders (*n* = 24) compared with CSF samples from healthy controls (*n* = 4100). They recorded an intrathecal immune response in 9 patients (4 with schizophrenia) in addition to blood-brain barrier dysfunction in 18 patients (9 of them with schizophrenia). Neopterin (a human macrophage-produced molecule by IFN-γ stimulation) concentrations increased in 14 patients (8 of them with schizophrenia). However, such augmentation of pro-inflammatory reactions did not lead to significantly detectable changes in tryptophan or kynurenine metabolites [43].

### Blood interleukins and kynurenine pathway dysregulation in schizophrenia

In patients with schizophrenia, the levels of T-helper (Th-) 1-specific IFN-γ and TNF-α, and Th2-related IL-6 were significantly higher in the plasma of the schizophrenic group (*n* = 71) compared to controls (*n* = 174) while mean plasma tryptophan concentration of the schizophrenic patients was significantly lower in contrast with controls. Moreover, the authors found that Th1-related IL-2 and Th2-specific IL-4 were significantly lower in patients’ plasma. However, plasma transforming growth factor (TGF-) β1 did not show statistically significant difference even though the mean value tended to be higher in schizophrenic patients [46].

In addition, after 6 weeks of treatment with four different atypical antipsychotics (risperidone, amisulpride, olanzapine, and aripiprazole), patients with schizophrenia exhibited a significant reduction of plasma IL-6 and TNF- α [23]. Interestingly, both mean plasma tryptophan and kynurenine levels were significantly increased after 6 weeks of antipsychotic treatment.

Finally, in patients with schizophrenia, the Th1/Th2 ratio (defined as [IFN-γ]/[IL-4]) showed a significant positive correlation with admission levels of plasma IL-6 and plasma TNF-α. This ratio also positively correlated with the tryptophan breakdown index (defined as plasma [kynurenine]/plasma [tryptophan]) [46].

According to a study conducted in patients with chronic schizophrenia (*n* = 51), compared with healthy control subjects (*n* = 45), the KYNA/3-HK ratio and IL-4 levels, but not soluble IL-2 receptor (sIL-2R) and IFN-α levels, were consistently decreased in schizophrenia patients at all analyzed time points (at time of admission, after a 4-week treatment and remission). Additionally, sIL-2R levels were positively correlated with the number of relapses before treatment [44].

The same study reported that in a subgroup of patients with poor response to pharmacotherapy, initial KYNA levels and the KYNA/3-HK ratio negatively correlated with the number of relapses. Positive association of sIL-2R levels with the number of relapses was also evident in this subgroup; those were treated 4 weeks later with clozapine. Finally, a negative correlation between IFN-α and 3-HK level (*r* = − 0.875) as well as a positive correlation between KYNA and IFN-α (*r* = 0.472) was shown [44].

On the other hand, one out of the 10 studies systematically reviewed here disclosed no statistically significant correlation between kynurenine pathway dysfunction and the presence of interleukins [45]. Despite a significantly higher KYN/tryptophan ratio in patients compared to healthy controls (patient, 0.055 ± 0.003; control, 0.048 ± 0.002; *t* = 2.02, df = 68, *p* < 0.05), no statistically significant relationships were revealed between IFN-γ values and the KYN/tryptophan ratio among the groups (*p* = 0.23). The differences of this ratio could not be associated with concomitant IFN-γ elevation. Therefore, this study suggests that this increased activity is not IDO mediated [29–31, 45].

### CSF interleukins and kynurenine pathway dysregulation in schizophrenia

The potential role of inflammation and kynurenic acid in psychotic disorders (mostly schizophrenia) was also investigated in twins [48]. Kegel and colleagues discovered a significant association between psychotic symptoms, interleukins, and kynurenine pathway metabolites (measured mostly in CSF) in Swedish-born, same-sex twins discordant for schizophrenia and other psychotic disorders [48]. In detail, they presented a relationship between IL-8 and QUIN (estimate 1.20, standard error [SE] 0.19, *t* = 6.49, *p* = 0.0001) and TNF-α and QUIN (estimate 155, SE 29.7, *t* = 5.21, *p* = 0.0006). By analyzing the intra-pair differences between twins, the authors have revealed associations between kynurenine metabolites and cytokines (QUIN, IL-8, and TNF-α) in complete twin pairs [48].

Another study by Schwieler and collaborators detected a significant correlation between IL-6 and KYNA-production in patients with schizophrenia reflected by the tryptophan to KYNA ratio (*r* = − 0.49; *p* = 0.024) [47].

Similarly, the authors observed a putative association between elevated IL-6 levels and KYNA in cultured fetal human cortical astrocytes exposed to recombinant human IL-6 (10 ng/mL). Increased levels of KYNA 48 h (1.55 ± 0.097 nM vs. 1.29 ± 0.054 nM, *p* = 0.038) and 72 h (1.83 ± 0.070 nM vs. 1.56 ± 0.053 nM, *p* = 0.045) after IL-6 stimulation were detected [47].

### Blood CRP and kynurenine pathway dysregulation in schizophrenia

Only one study exclusively investigated CRP as an inflammatory marker and a possible kynurenine pathway deregulation in acutely ill inpatients with major depressive disorder (MDD) (*N* = 35), bipolar disorder (BD) (*N* = 53), schizoaffective disorder (SZA) (*N* = 40), and schizophrenia (*n* = 21) versus healthy controls (*N* = 92).

Serum KYNA and/or KYNA/QUIN were significantly reduced in all patient subgroups versus healthy controls except for the schizophrenia subgroup, which did not differ from healthy controls [49].

Moreover, a post hoc comparison of patients divided into the categories of non-psychotic affective disorder, affective psychosis, and psychotic disorder (non-affective) manifested reduced levels of KYNA in both the affective disorder and affective psychosis groups. KYNA/3HK was significantly reduced in affective psychosis in comparison to non-psychotic affective disorder and in affective psychosis when compared to healthy controls. Similarly, KYNA/QUIN differed across groups and was significantly reduced in the affective disorder and affective psychosis groups compared with healthy controls. However, no significant difference was shown for patients with schizophrenia.

Follow-up *t* tests showed that healthy controls had significantly lower CRP concentrations or a statistical trend towards lower CRP concentrations than the MDD, BD, and SZA subgroups. Nonetheless, this was not the case of the patient subgroup with schizophrenia [49].

## Discussion

The results of this study highlight correlations between inflammation, the kynurenine pathway, and schizophrenia in the available literature of human research from the last 10 years. Although few obtained studies related to the specific topic and methodological differences existed between studies, especially concerning sample collection (plasma or CSF), correlations were found between the three mentioned variables.

In regard to studies with only serum samples, correlations between [IFN-γ]/[IL-4] and [kynurenine]/[tryptophan] were found in schizophrenia. Other studies with schizophrenic patients reported correlations between IFN-α and 3-HK levels, as well as correlations between KYNA and IFN-α levels. Additionally, other selected studies found correlations between IgA-mediated responses with levels of tryptophan metabolites (i.e., kynurenine pathway) in schizophrenic patients. Nevertheless, we did not find evidence for a relationship between CRP and tryptophan metabolite levels in patients with schizophrenia.

Regarding studies with only CSF samples, correlations between IL-8 and QUIN, TNF-α and QUIN, and IL-6 and KYNA production were also found in schizophrenia. However, one study demonstrated augmented pro-inflammatory reactions but without detection of tryptophan metabolites using CSF-cytometry of schizophrenic patients. Figure 1 helps to give a summary of the principal findings on the inflammation effects on the kynurenine pathway in schizophrenia.

### Cytokines, kynurenine pathway, and schizophrenia

Since the discovery of cytokines, our knowledge of signal transmission between cells has been dramatically expanded [30, 50]. Cytokines have been initially described in systemic inflammatory diseases. Newer evidence suggests that they are also related to other disorders like schizophrenia. The results of the present systematic review revealed the following cytokines as possible candidates for deregulatory mechanisms on the kynurenine pathway in schizophrenia: TNF-α, IFN-γ, IL-6, IL-4, IL-8, and IFN-α.

#### TNF-α

The results showed a correlation between higher TNF-α values and augmented QUIN concentrations in schizophrenia. TNF-α is known for its effect on microglial cells, generating a strong diffuse pro-inflammatory response in the CNS [51]. In addition to that, authors have postulated in the last years that TNF-α modifies the metabolic pathway of tryptophan producing higher quantities of neuroactive substances (i.e., KYNA and QUIN) in the CNS [29–31]. The result of an unbalanced QUIN production could eventually explain cognitive impairment disorders, a clinical entity closely connected to schizophrenia [4, 52, 53].

#### IFN-γ

The obtained results from this review between IFN-γ and kynurenine pathway were ambiguous. It is known that IFN-γ activates Th1 cells promoting cytotoxic cell activity and immune responses. Tryptophan catabolism towards KYN formation is mostly mediated by IDO, which is believed to be activated by an increased concentration of IFN-γ [54]. In schizophrenia, authors have also postulated an effect of IFN-γ on the blockade of NMDA and alpha-7-n-acetylcholinergic receptors through an increase of KYNA concentrations [55, 56]. Moreover, different studies relate IFN-γ with an increase of 3-HK in patients with schizophrenia [57–59]. However, the studies from this systematic review reported two contradictory results. On the one hand, one included study evidenced significant correlations between IFN-γ/IL-4 in plasma and tryptophan breakdown ratio ([kynurenine]/[tryptophan]) in schizophrenia. On the other hand, another included study showed no significant relationships between IFN-γ values and the kynurenine/tryptophan ratio. Based on the post-mortem study findings from Miller et al., we suggest that IDO expression does not respond to a concomitant augmentation of IFN-γ in the CNS and that IFN-γ eventually has a minor effect on the kynurenine pathway in schizophrenia [60].

#### IFN-α

The results from this systematic review pointed out a negative correlation between IFN-α and 3-HK but a positive one with KYNA in schizophrenia. Known functions of IFN-α include the stimulation of macrophage antibody-dependent cytotoxicity [51]. Microglia are phagocytic, macrophage-like cells in the CNS and represent the main portion of active innate immune defense in the CNS [61]. Both overactivation and administration of IFN-α induce higher production of KYNA, which can be neurotoxic. Although this effect was published for depressive patients, the augmented concentration of IFN-α was shown to unbalance neurophysiological mechanisms also in subjects with schizophrenia [62].

#### IL-6

The results of this study found that elevated IL-6 levels correlated with higher KYNA values in schizophrenia. This cytokine exerts an effect on different pro-inflammatory mechanisms in microglial cells [50, 51] like microglia priming—i.e., the transition from resting to activated state [63]; regulation of the inflammatory response [64]; and overproduction of IL-6 in the aged brain [65]. Due to these microglial functions, authors pointed out that IL-6 was associated with KYNA production by increasing this metabolite in patients with schizophrenia [19]. Other major functions of IL-6 are also the synthesis and promotion of cortisol by hypothalamic stimulation [66]. While such IL-6-action may be related to some other psychiatric disorders like unipolar depression [67], elevation of cortisol production by IL-6-action could also be linked to schizophrenia [68–70]. We suggest that this increased production of cortisol by IL-6-action could be provoked due to two factors: the first one, the exposure to physical or environmental stress, and the second one, genetic vulnerability (i.e., the interaction between these stressors and risk alleles [68]). Both factors, i.e., stress and genetic vulnerability, are components of the mentioned vulnerability-stress model [4, 71, 72].

#### IL-8

The present review shows that this chemokine of the CXC family is related to the kynurenine pathway in schizophrenia. IL-8 contributes to activation and recruitment of neutrophil granulocytes but was also shown to increase QUIN production, as different Alzheimer’s disease studies in human post-mortem and serum samples have shown [50, 52]. IL-8-induced QUIN induction may be involved in microglial and neuronal activation/damage in the hippocampus eventually contributing to cognitive dysfunction [52]. Although supporting data has been obtained in models designed for Alzheimer’s disease research, overproduction of IL-8 and its relation with schizophrenia could also explain cognitive impairment symptoms in patients with schizophrenia [52]. Finally, in vitro studies demonstrated that the combination of TNF-α/IFN-γ promotes an augmentation of IL-8 in human astrocytes [73, 74]. Nevertheless, the present review found no significant correlations with IFN-γ and positive correlations with TNF-α and kynurenine pathway in patients with schizophrenia.

#### IL-4

Only one cytokine from the γ-chain-cytokine family, namely IL-4, was related to the kynurenine pathway in schizophrenia. It is known that this cytokine is associated with Th2 responses, but it also suppresses KYNA production through blockade of the KAT2 [50, 75]. The results of the present systematic review highlight that patients with schizophrenia seem to show lower plasma concentration of IL-4. This might lead to an imbalance due to a lower Th2 response and could eventually explain the augmentation of KYNA production in schizophrenia, as previously shown in an animal model for Alzheimer’s disease [75].

Interestingly, these results contradict the hypothesis of a predominant Th2 response in schizophrenia and rather support a shift of the Th cells towards a Th1 response [72, 76].

### Immunoglobulins, kynurenine pathway, and schizophrenia

The present review also shows a relationship between IgA-mediated responses and schizophrenia. This antibody plays a crucial role in the immune function of mucous membranes mostly in the gastrointestinal tract providing protection against microbes [77]. Our assessed studies revealed strong correlations between an IgA-mediated response and tryptophan metabolites, especially KYNA and QUIN. Thus, the inflammatory phenomenon most likely unbalances the tryptophan metabolism subsequently contributing to psychotic symptoms.

### Negative findings: CRP and CSF-cytometry influence on the kynurenine pathway and schizophrenia

Our review did not show any significant correlation between CRP and kynurenine metabolism. However, other studies have reported an association between CRP levels and schizophrenia severity [78]. While cytometry of CSF revealed a macrophage-induced pro-inflammatory reaction (i.e., increased neopterin levels) in patients with schizophrenia, a relationship with KYNA or tryptophan metabolites was not found.

### Cellular substrates regulating interactions between kynurenine pathway and inflammation

#### Cellular regulation of kynurenine pathway

Overactivation of astrocytes in schizophrenia was shown to be independent of the patients’ medication state [79]. This increased activity of astrocytes in schizophrenia and the persistent reduction of microglial KMO activity result in increased kynurenic acid formation in astrocytes [80]. This finding is consistent with CSF investigation by Schwieler et al. 2015 that showed a positive correlation between IL-6 and KYNA. However, Szymona et al. found decreased KYNA/3-HK ratio in the blood. This could be explained by the KYNA inability to cross the blood-brain barrier.

It is noteworthy that the activation of astrocytes may further facilitate the catabolism of QUIN by QPRTase. However, a recent review postulated that in some cases, kynurenine pathway metabolism is likely to be rerouted from KYNA to QUIN production in brain tissue in states of inflammation with microglial activation and macrophage infiltration [81]. Microglial activation was only found in a small percentage of schizophrenia patients and seems to be more pronounced in chronic schizophrenia patients [79]. This might underlie the activation of QUIN pathway in CSF study by Kegel et al. 2017 [48].

#### Kynurenine pathway and inflammation

The interaction between intracellular kynurenine pathway and the inflammatory process seems to be bidirectional and more complicated. The first direction is demonstrated by several previous studies showing that inflammatory cytokines can dramatically affect the astrocyte and microglial activity and thus the production of KYNA and QUIN. For example, in vitro studies reveal enhanced IDO-messenger RNA-expression in microglia following IFN-γ stimulation, and to a lesser extent in astrocytes [23]. Both IFN-γ and TNF-α increase also the production of QUIN by microglia and macrophages [23, 82]. These effects could explain the positive findings found in blood [44–46] and the correlation between TNF-α and QUIN in CSF [48].

In contrast, the opposite direction implies that some kynurenine metabolites can modulate the inflammatory processes. At excitotoxic concentrations, QUIN induces the expression of several pro-inflammatory cytokines and chemokines such as IL-1β, monocyte chemotactic protein 1, and IL-8 in astrocytes [83]. KYNA, an NMDA receptor antagonist, has been shown to act as an immunosuppressant in mice. Such antagonism caused Th1 effector cells to produce less IL-2 and IFN-γ, whereas it affected Th2 cells to produce more interleukin (IL-) 10 and IL-13. As proposed by a recent review, this interaction suggests a kynurenine pathway-immune feedback loop that may be disrupted in schizophrenia [81]. We suggest that the competition between the pro-inflammatory QUIN (an NMDA receptor agonist produced by microglia) and the immunosuppressant KYNA (an NMDA receptor antagonist) may determine the inflammatory status and/or the clinical picture of certain psychiatric diseases related with neuroinflammation.

#### Intercellular interactions

Regarding the intercellular communication, astrocytes produce neuropeptide Y which inhibits microglial activity. In that sense, astrocytes may inhibit pro-inflammatory cytokine-induced IDO and the microglial kynurenine pathway. In addition, microglial cells mainly secrete type-1 cytokines (e.g., IL-12), while astrocytes inhibit the production of pro-inflammatory cytokines and secrete type-2 cytokines (e.g., IL-10).

In the light of our results, in schizophrenic patients, such equilibrium between type 1 and type 2 response in the CNS is disturbed, as showed in the study of Kim et al. [46] and included in this review. It seems that there is an imbalance between the activation of microglial cells and astrocytes [79]. This particular unbalance of the intercellular communication between astrocytes (producing neuroprotective KYNA and PIC) and microglia (producing neurotoxic QUIN) could suggest a competitive interaction between both of them, where the different activation patterns of microglial cells and astrocytes may underlie the biological variations among schizophrenic states.

### Limitations and future directions

#### Limitations

Some limitations have to be taken into account for the present systematic review. First of all, to fulfill our primary aim to find studies assessing the correlation between the “kynurenine pathway,” “inflammation,” and “schizophrenia,” the used Booleans restricted the results; we could not find many studies with other biomarkers, such as enzymes. Nevertheless, this approach enabled us to specifically address the correlations between the three-targeted variables. Second, the studies included in our sample, although they delivered mostly significant results, appeared to be heterogeneous regarding the methodology and patient selection criteria. Third, the definition of schizophrenia varied between studies as it was amenable to the subjectivity of clinical inspection. Independently of the similarities between ICD and DSM classification, standardized protocols to select patients with schizophrenia do not exist and, therefore, cannot guarantee high homogeneity of samples between studies. Finally, some studies did not pay attention to some confounding factors or variables such as duration of illness, doses of antipsychotic drugs, symptom severity, or smoking potentially influencing some of the reviewed results here.

#### Future directions

This current article focuses mostly on the influence of inflammation in the tryptophan metabolism in human subjects with schizophrenia. Due to the lack of data on this matter, future studies that investigate the influence of inflammation in the CNS especially related to the kynurenine pathway in schizophrenia are warranted. To integrate different pathways and mechanisms will allow for the possible identification of therapeutic targets and interventions for handling and preventing relapses. While there are differences between studies, the literature overall suggests significant correlations between these three variables.

These findings raise many questions, such as “what are the most important metabolites that relay inflammation or inflammatory markers and the kynurenine pathway in schizophrenia?” For example, in an animal model of pregnant rats, the deletion of one allele for KMO (KMO^+/−^) in the maternal genetic material brought to a disproportionate KYNA increase in the brain of KMO^+/−^ offspring rats, compared to KMO wild-type offspring rats (KMO^+/+^) [84]. As mentioned before, in subjects with schizophrenia, the overproduction of KYNA contributes mainly to an imbalance of Th1/Th2 responses. Additionally, in other mouse schizophrenia model, the dysregulation of KYNA explained different cognitive impairments such as anticipatory responses and reduced locomotor activity [85]. The search for understanding this mechanism also in humans, mostly in patients with schizophrenia, will allow to conduct future studies that could regulate the KMO-genetic expression and finally to avoid the imbalance between KNYA and QUIN mechanisms, as showed in patients with schizophrenia.

Additionally, the regulation of NMDA receptors could also have positive effects on schizophrenia, since in the case of schizophrenia KYNA is overexpressed and involved in the receptor antagonism, as well as in the imbalance of inflammatory responses. An experimental model in mice has shown that the regulation of NMDA receptors through KAT2 inhibitors attenuates the KYNA response in the pre-frontal cortex [86]. The prevention of KYNA and the regulation of glutamate release could contribute to the treatment of schizophrenia, mostly to cognitive deficits associated with this mental disorder [86]. Additionally, the regulation of the enzymes related to the kynurenine pathway has suggested to have positive results as an immunomodulatory therapy [87]. Although this data is shown in other disease models, such as depression [87], the stabilization of immune responses in schizophrenia could also contribute to a posterior regulation of noxious effects of the kynurenine pathway, as described before.

What actual therapeutic choices/options could we have in schizophrenia, where a predominant inflammatory factor alters the homeostasis of nerve cells? Currently and to the best of our knowledge, there is no evidence supporting a specific and direct therapeutic option that could regulate the effects of pro-inflammatory cytokines in the kynurenine pathway. What we already know is that some antipsychotics can partially modulate the immune imbalance and the overproduction of KYNA, a natural NMDA receptor antagonist [88]. According to the experimental studies in mice, it is shown that olanzapine regulates the activation of IDO [89]. Nevertheless, the only information we have is related to depression and models for therapeutic targets in schizophrenia regarding this matter are needed. Finally, anti-inflammatory drugs (i.e., non-steroid anti-inflammatory drugs) as add-on therapy to antipsychotic therapy have shown regulating effects on psychotic symptomatology, compared to antipsychotic therapy only [88, 90]. However, the detailed pharmacological effects of the add-on anti-inflammatory therapy in schizophrenia remain unknown, and most evidence regarding this topic is inconclusive and, due to the amount of included studies, limited [91]. In this case, more studies are needed to comprehend how does the pharmacology of anti-inflammatory drugs work and to understand if these drugs, by inhibiting inflammatory agents, could indirectly or directly influence the kynurenine pathway in schizophrenia.

## Conclusions

In conclusion, the present systematic review found a relationship of elevated IL-6, IL-8, and TNF-α concentrations with the kynurenine pathway in schizophrenia. These higher values could be an explanation for the psychotic symptomatology and cognitive disturbances. However, this systematic review did not find a correlation between CRP, CSF-cytometry, IFN-γ, and the kynurenine pathway in schizophrenia. With respect to the Th response and lower IL-4 concentrations, we conclude that overactivation of the kynurenine pathway is mostly related to a reduced Th2 and augmented Th1 response. Overall, we conclude that there is a strong immunoglobulin-mediated response against metabolites of the kynurenine pathway, contributing as well to inflammatory mechanisms.

While different methodologies have been applied in the included studies and the results show some heterogeneity, our systematic review gives support to a picture of schizophrenia that integrates inflammatory mechanisms and nerve cell physiology. To expand the understanding of this matter, further studies should be performed.

## Data Availability

The data that support the findings of this study are available from the corresponding author on request.

## Abbreviations

3HK: 3-OH-kynurenine
AA: Anthranilic acid
ACMSD: Aminocarboxymuconate semialdehyde decarboxylase
Af: Affective disorder
BPRS: The Brief Psychiatric Rating Scale
CANTAB: The Cambridge Neuropsychological Test Automated Battery
CDSS: Calgary Depression Scale for Schizophrenia
CNS: Central nervous system
CRP: C-reactive protein
CSF: Cerebrospinal fluid
DSM-IV-TR: Diagnostic and Statistical Manual of Mental Disorders-Text Revision
DZ: Dizygotic twins
FF: Fibromyalgia and Chronic Fatigue Syndrome Rating Scale
GAF: Global Assessment of Functioning Scale
HAM-A: Hamilton Anxiety Rating Scale
HAM-D: Hamilton Depression Rating Scale
ICD 10: The International Classification of Diseases Tenth Edition
IDO: Indoleamine 2,3-dioxygenase
IFN-: Interferon-
Ig: Immunoglobulin
IL-: Interleukin-
KAT: Kynurenine aminotransferase
KMO: Kynurenine 3-monooxygenase
KYN: Kynurenine
KYNA: Kynurenic acid
MINI: Mini-International Neuropsychiatric Interview
MDD: Major depressive disorder
MZ: Monozygotic twins
ND: Not determined
NMDA: *N*-methyl-d-aspartate
PANSS: The Positive and Negative Syndrome Scale
PIC: Picolinic acid
QPRTase: Quinolinate phosphoribosyltransferase
QUIN: Quinolinic acid
SANS: Scale for the Assessment of Negative Symptoms
SAPS: Scale for the Assessment of Positive Symptoms
SCID-I/II for DSM-IV: Structured Clinical Interview for Diagnostic and Statistical Manual IV Axis I/II Disorders
SDS: Schedule for Deficit Syndrome
sIL-2R: Soluble interleukin-2 receptor
SPQ-B: Schizotypal Personality Questionnaire Brief
Sz: Schizophrenia
SzA: Schizo-affective disorder
TDO: Tryptophan 2,3-dioxygenase
TGF-: Transforming growth factor
Th: T-helper
TNF­α: Tumor necrosis factor­α
Trp: Tryptophan
TRYCATs: Tryptophan catabolites
XA: Xanthurenic acid
YMRS: The Young Mania Rating Scale
